# Concurrent Use of Anifrolumab and Belimumab in a Patient With Systemic Lupus Erythematosus Presenting With Recurrent Severe Cutaneous Involvement and Lupus Nephritis

**DOI:** 10.7759/cureus.97252

**Published:** 2025-11-19

**Authors:** Maria Dolores Manjón-Rodríguez, Francisco J Borrego-Utiel, Enoc Merino-García, Alba Gil-Morillas, Agustín Colodro-Ruiz

**Affiliations:** 1 Internal Medicine, Hospital Universitario de Jaén, Jaén, ESP; 2 Nephrology, Hospital Universitario de Jaén, Jaén, ESP

**Keywords:** anifrolumab, b-cell activating factor, belimumab, cutaneous lupus erythematosus, lupus nephritis, systemic lupus erythematosus, type 1 interferon

## Abstract

Anifrolumab and belimumab are two biologic agents extensively employed in patients with systemic lupus erythematosus (SLE). Anifrolumab has demonstrated pronounced efficacy in reducing cutaneous inflammatory activity in cases refractory to other therapies. Belimumab has exhibited clinical effectiveness in ameliorating articular symptoms and renal involvement, as well as in decreasing the occurrence of new disease flares. The case presented here constitutes a singular report in the literature owing to the combined use of both biologic therapies. The patient initially developed intense inflammatory facial lesions, which responded completely to anifrolumab. Thereafter, lupus nephritis developed and was managed with belimumab following cessation of anifrolumab, leading to improvement in renal parameters. Upon reactivation of cutaneous lesions, anifrolumab was reinstated concomitantly with belimumab, resulting in full resolution of the skin lesions while preserving complete remission of the lupus nephritis. We discuss how various biologic agents can act on separate pathogenic pathways contributing to organ involvement in SLE. We suggest that in some cases with severe cutaneous and renal involvement that do not respond to anifrolumab and belimumab individually, the agents may be used in combination to achieve adequate control of lupus disease.

## Introduction

Systemic lupus erythematosus (SLE) is a complex disease with highly variable clinical expression throughout the patient’s life. Both innate and adaptive immunity are involved in organ damage, which can influence the widely varying responses of patients to immunosuppressive therapies [1].

Anifrolumab is a monoclonal antibody directed against the type 1 interferon (IFN-1) receptor that inhibits this signaling on the innate and adaptive immune responses and local inflammatory mechanisms, all of which are involved in the pathogenesis of SLE. Its efficacy in mucocutaneous lesions has been demonstrated in the TULIP trials and subsequently in real-life studies [2-5]. Belimumab is another monoclonal antibody that acts on adaptive immunity by inhibiting B-cell activating factor (BAFF), which reduces the survival of autoreactive B cells, promoting their apoptosis and decreasing the generation of memory B cells and plasma cells and the production of autoantibodies, the main pathway involved in renal lesions [6]. Its efficacy on renal lesions has been demonstrated in the BLISS-LN trial and in subsequent real-life studies [7,8].

The phenotype of patients with SLE can change over time, such that between 4% and 40% of patients with cutaneous lupus may subsequently develop systemic involvement [9], often requiring a change in immunosuppression if an adequate response is not achieved with the medication being used at that time. Whereas the pathogenesis of cutaneous lesions involves primarily the local production of IFN-1 and local inflammatory mechanisms intrinsic to innate immunity, renal damage depends more heavily on the generation and survival of B lymphocytes facilitated by BAFF, and on the production of systemic autoantibodies that subsequently deposit in the kidney, activate complement, and, ultimately, lead to renal damage [10-12]. This shift in immunological profile would therefore justify a change in the immunosuppressive treatment strategy to block different pathogenic mechanisms. However, if the initial improvement achieved with one therapy is lost after switching, the need to use both agents in combination to control damage in both organs may arise.

We report a case of SLE with initial cutaneous involvement followed by renal disease, in which combined treatment with anifrolumab and belimumab was required to achieve adequate disease control.

## Case presentation

A 23-year-old woman was diagnosed with SLE in June 2023 based on clinical presentation (fever, arthralgia, cutaneous lesions, anemia, lymphopenia) and a positive antinuclear antibody (ANA) titer of 1:1,280, with a SLEDAI-2K score of 7. Anti-dsDNA was >400 IU/mL, with reduced complement levels (C3 = 63 mg/dL, C4 = 6.6 mg/dL) and no albuminuria (16.2 mg/g). Treatment was initiated with hydroxychloroquine 200 mg/day and prednisone 15 mg/day. Azathioprine 50 mg/day was added in October 2023, but was discontinued two months later due to hypertransaminasemia, a well-known complication of this medication [13].

**Figure 1:**
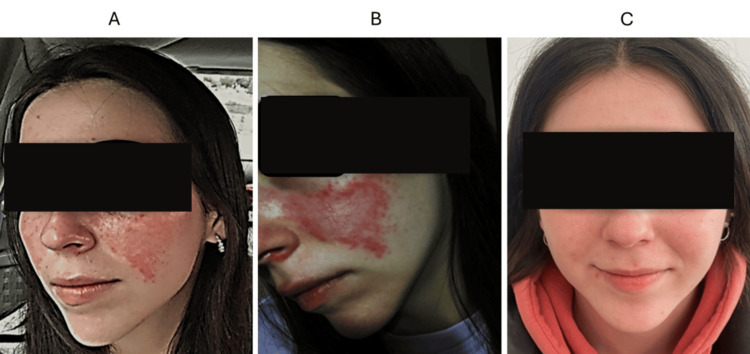
Discoid lupus lesions on the face during the flare in January 2024 before treatment with anifrolumab (A). Discoid lupus lesions in September 2024 after discontinuation of anifrolumab and initiation of belimumab (B). Resolution of cutaneous lesions after two months of combined treatment with anifrolumab and belimumab (C).

In January 2024, the patient presented with a more severe cutaneous flare, characterized by well-demarcated, intensely erythematous lesions with mild superficial scaling, involving both malar cheeks, the nasal bridge, and the scalp (CLASI-A 6) (Figure 1, Panel A), oral ulcers, severe arthralgia, and marked functional impairment. Laboratory tests at this time showed a similar profile (anti-dsDNA = >400, C3 = 75 mg/dL, C4 = 5.1 mg/dL). Consequently, in February 2024, three intravenous (IV) pulses of methylprednisolone (250 mg each) were administered, and treatment with anifrolumab 300 mg IV/month was started, in combination with hydroxychloroquine 200 mg/day, prednisone 15 mg/day, and mycophenolate mofetil (MMF), initially at 500 mg/day and increased to 1 g/day one month later.

After two months with anifrolumab, the patient exhibited complete resolution of cutaneous lesions with normalization of complement levels (C3 = 125 mg/dL, C4 = 12.6 mg/dL), allowing a reduction of prednisone dose to 12.5 mg/day. However, anti-dsDNA titers remained elevated (>400 IU/mL), and mild albuminuria (156 mg/g) was detected, accompanied by an active urinary sediment (10-20 erythrocytes/hpf).

One month later (May 2024), the patient remained asymptomatic; however, proteinuria increased (Table 1). Hence, a nephrology consultation was requested, and anifrolumab was continued until July 30, 2024. A renal biopsy was performed in August 2024, revealing the following findings: 28 glomeruli, of which 17 showed mesangial hypercellularity, three (10%) with fibrocellular crescents, endocapillary hypercellularity in one, segmental sclerosis in six, and capsular adhesions in six. Interstitial fibrosis was observed in 10% of the sample. Immunofluorescence demonstrated granular mesangial and membranous deposits of IgA 3+, IgG 2+, IgM 2+, C3 3+, and C1q 2+. A diagnosis of diffuse proliferative lupus nephritis was established (activity index = 3/24, chronicity index = 1/12). The dose of MMF was increased to 2 g/day, prednisone was maintained at 10 mg/day, and belimumab (560 mg = 10 mg/kg IV monthly) was initiated.

**Table 1:** Analytical progression before and after initiation of belimumab.

	April 2024	May 2024	August 2024	Month +1	Month +2	Month +3	Month +5	Reference values
Creatinine (mg/dL)	0.58		0.64		0.63	0.63	0.71	0.5–0.9
Proteinuria (mg/g)		987	1099	312	162	97	103.6	<150
Albuminuria (mg/g)	462	285	786	188	117	49.2	32.6	<30
Erythrocytes in urine (cells/hpf)	3–5	10–20	20–40	20–40	10–20	0	0	0–5
ANA titers	-	-	1/640	-	-	-	-	Negative
Anti-dsDNA (UI/mL)	>400		352		31.5	27	62	0–10
C3 (mg/dL)	95	-	98	-	99	92	-	75–180
C4 (mg/dL)	11.1	-	15.7	-	15.2	10.9	-	10–40

In October 2024, the patient complained of a recurrence of similar cutaneous lesions on the face and scalp (Figure 1, Panel B), along with significant arthralgia. Laboratory tests showed a marked reduction in proteinuria, though microhematuria persisted (Table 1). Given the severity of the cutaneous involvement, anifrolumab (300 mg IV monthly) was reintroduced, maintaining treatment with belimumab, MMF 2 g/day. Prednisone was initially increased to 30 mg/day but was rapidly tapered to 10 mg/day within one month.

After two months of anifrolumab and 3 months of belimumab, cutaneous involvement and arthralgias had resolved (CLASI-A score 0 (Figure 1, Panel C). Laboratory tests demonstrated complete remission of proteinuria, normalization of urinary sediment, a reduction in anti-dsDNA titres, and normalization of complement levels (Table 1).

After 10 months of treatment, the patient remains asymptomatic, without skin involvement or proteinuria, and has not experienced any infections or medication-related toxicity. The current treatment includes deflazacort 6 mg/day, MMF 2 g/day, and subcutaneous belimumab 200 mg/week and anifrolumab administered monthly.

## Discussion

The case presented here is noteworthy for the combined use of anifrolumab and belimumab in the same patient, illustrating the distinct pathogenic mechanisms involved in cutaneous and renal involvement in SLE. The skin is rich in plasmacytoid dendritic cells, which can capture a wide variety of antigens entering through the skin, including self-DNA released from damaged cells and leucocytes. This triggers IFN-1 release, which, in turn, stimulates autoantibody production by B cells, leading to the formation of in situ immune complexes and keratinocyte damage. Keratinocytes themselves respond to inflammatory damage by producing IFN-1 [10]. This response is less dependent on systemic autoantibodies and is typically associated with minimal complement activation.

In contrast, lupus nephritis is more strongly linked to the adaptive immune response, with sustained activation of autoreactive B lymphocytes in lymphoid tissues and within the kidney, leading to continuous production of specific autoantibodies (anti-dsDNA, anti-C1q, anti-nucleosome). These antibodies form circulating immune complexes that subsequently deposit in the kidney, triggering complement activation and initiating the full cascade of tissue injury. Although IFN-1 may modulate B- and T-cell responses, the survival of autoreactive B cells, generation of memory B cells and plasma cells via the BAFF pathway, and phenotypic switching of T cells appear to be the primary drivers of renal damage [11,12].

Clinical trials and real-world experience have shown that anifrolumab and belimumab may exert differing effects on organ involvement in SLE. Anifrolumab clearly improves the inflammatory component of cutaneous lesions within as little as four weeks, as evidenced in the TULIP-1 and TULIP-2 trials [2,5], and has also proven effective in real-world settings in patients refractory to corticosteroids and other immunosuppressants [14], including those in whom belimumab was replaced by anifrolumab due to poor response [15,16]. Conversely, anifrolumab has not shown significant benefit in lupus nephritis, even at higher doses than those used in our patient [17]. Belimumab, in contrast, has demonstrated clinical efficacy in lupus nephritis, including a reduction in the incidence of new renal flares [7,8]. Our case supports these mechanistic differences in organ-specific pathogenesis, supported by the following sequence: rapid improvement of cutaneous lesions with anifrolumab; onset of proteinuria despite ongoing anifrolumab treatment; renal improvement with belimumab; recurrence of cutaneous lesions after anifrolumab withdrawal; and resolution of lesions after its reintroduction.

The development of renal involvement during anifrolumab therapy in our patient raises the question of whether the renal flare was triggered by this biological treatment. Bao et al. [16] also reported two cases with increased proteinuria following anifrolumab administration. In one case, proteinuria appeared after treatment initiation and improved upon discontinuation. In the other, baseline mild proteinuria worsened during treatment, prompting a renal biopsy, but followed by improvement in proteinuria after three months of continued anifrolumab. No specific mechanisms have been identified whereby IFN-1 pathway inhibition might exacerbate renal immunopathology. It is therefore plausible that, in patients with severe SLE, cutaneous involvement may present first, with renal manifestations developing later despite immunosuppressive therapy. Indeed, several studies and clinical trials have reported renal flares occurring during appropriate immunosuppressive treatment [18].

The reasons underlying phenotypic shifts in SLE over time remain unclear. Cumulative environmental exposures (e.g., ultraviolet radiation, infections, oxidative stress), hormonal and epigenetic factors, and immunosenescence are among the proposed contributors to the evolution of immune responses and resultant changes in autoantibody profiles, leading to variable systemic involvement [19]. The presence of anti-dsDNA and anti-Sm antibodies has been identified as a risk factor for the progression of cutaneous-dominant SLE to systemic damage [9,18,20]. In our case, high anti-dsDNA titers from the beginning suggest that renal pathogenic mechanisms were already active, and the eventual development of lupus nephritis may represent the natural progression of the disease.

The response of cutaneous lesions and serological markers to anifrolumab was greater and more sustained in patients with a high IFN-1 signature, as seen in the TULIP trials [5]. In our patient, the IFN-1 signature and BAFF levels could not be assessed. In this context, the development of reliable biomarkers for use in routine clinical practice could allow early identification of the immunological pathways driving organ involvement at any given time, predicting a change in phenotype and allowing the most appropriate treatment to be chosen [21,22].

## Conclusions

The combined use of anifrolumab and belimumab could be considered in patients with concurrent cutaneous and renal involvement in whom each agent used individually has been ineffective. Nevertheless, the clinical utility of this combination should be investigated in future studies to demonstrate its efficacy and potential adverse effects. The distinct immunopathological mechanisms underlying SLE-related organ damage may guide the tailoring of treatment to the patient’s phenotype, particularly when that phenotype evolves over time. The identification and implementation of pathway-specific biomarkers would allow the advancement of precision medicine in SLE, enabling clinicians to select the most effective therapeutic strategies for each individual patient.
